# Alcohol as a Non-UV Social-Environmental Risk Factor for Melanoma

**DOI:** 10.3390/cancers14205010

**Published:** 2022-10-13

**Authors:** Takeshi Yamauchi, Sarah Shangraw, Zili Zhai, Dinoop Ravindran Menon, Nisha Batta, Robert P. Dellavalle, Mayumi Fujita

**Affiliations:** 1 Department of Dermatology, University of Colorado Anschutz Medical Campus, Aurora, CO 80045, USA; 2 Department of Veterans Affairs Medical Center, VA Eastern Colorado Health Care System, Aurora, CO 80045, USA; 3 Department of Epidemiology, Colorado School of Public Health, University of Colorado Anschutz Medical Campus, Aurora, CO 80045, USA; 4 Department of Immunology and Microbiology, University of Colorado Anschutz Medical Campus, Aurora, CO 80045, USA

**Keywords:** melanoma, ethanol metabolism, alcohol, ethanol, acetaldehyde, alcohol dehydrogenase, aldehyde dehydrogenase, ultraviolet radiation

## Abstract

**Simple Summary:**

Malignant melanoma is an aggressive cancer of the skin and the leading cause of death from skin cancer. One major risk factor linked to melanoma development is exposure to UV radiation. However, the sharp increase in melanoma cases cannot be explained only by more UV exposure. Identifying additional modifiable social-environmental risk factors for melanoma beyond UV exposure would greatly impact public health initiatives and the methods of patient outreach and education. Recent studies have shown the link between melanoma and alcohol consumption. This perspective review paper aims to understand the mechanisms underlying ethanol’s ability to induce human cancers, including melanoma.

**Abstract:**

Although cancer mortality has declined among the general population, the incidence of melanoma continues to rise. While identifying high-risk cohorts with genetic risk factors improves public health initiatives and clinical care management, recognizing modifiable risk factors such as social-environmental risk factors would also affect the methods of patient outreach and education. One major modifiable social-environmental risk factor associated with melanoma is ultraviolet (UV) radiation. However, not all forms of melanoma are correlated with sun exposure or occur in sun-exposed areas. Additionally, UV exposure is rarely associated with tumor progression. Another social-environmental factor, pregnancy, does not explain the sharply increased incidence of melanoma. Recent studies have demonstrated that alcohol consumption is positively linked with an increased risk of cancers, including melanoma. This perspective review paper summarizes epidemiological data correlating melanoma incidence with alcohol consumption, describes the biochemical mechanisms of ethanol metabolism, and discusses how ethanol and ethanol metabolites contribute to human cancer, including melanoma.

## 1. Introduction

Cutaneous melanoma is an aggressive malignancy of the skin and a significant public health concern. Melanoma incidence continues to rise globally, placing a greater burden on industrialized countries [1,2,3,4,5]. In the United States, melanoma remains the fifth most common cancer, making up 6 and 5 percent of all new cancers in males and females, respectively [6,7]. Although melanoma mortality decreased from 2017 [8] to 2021 [9], the incidence continues to increase: approximately 99,780 new melanoma cases will be diagnosed in the United States in 2022 [6], almost doubling from 53,600 in 2002 [10]. The increased incidence of cutaneous melanoma has been hypothesized to be, in part, the result of improved screening and early detection. However, the incidence of advanced melanoma is also increasing, suggesting that risk factors for melanoma are on the rise [11,12,13].

Risk factors and conditions for the development of melanoma are categorized into three: (1) genetic risk factors, (2) phenotypic risk factors reflecting gene/environment interactions, and (3) social-environmental risk factors [14]. Genetic risk factors include family history, light skin/hair/eye color, DNA repair defects, and several melanoma risk genes, such as cyclin-dependent kinase (CDK) inhibitor 2A (*CDKN2A*), *CDK4*, BRCA1-associated protein-1 (*BAP1*), protection of telomeres 1 (*POT1*), and telomerase reverse transcriptase (*TERT*) [15,16]. Mutations in these tumor suppressor genes confer high susceptibility to melanoma. In contrast, some genetic factors, especially when interacting with phenotypic and environmental risk factors, have great significance in melanoma susceptibility. For example, melanocortin 1 receptor (*MC1R*) R (D84E, R142H, R151C, I155T, R160W, D294H) variants are associated with the fair skin and red hair color phenotype, which is prone to sunburn and has an increased risk of melanoma [15,16]. Phenotypic expressions of gene/environment interactions also include a personal history of skin cancer and numerous nevi [17,18,19,20,21,22]. While identifying these high-risk cohorts improves public health and clinical care management, genetic factors are unchangeable. Therefore, recognizing modifiable risk factors, such as social-environmental risk factors, is crucial from the clinical perspectives of patient outreach, education, and disease management.

The most notable social-environmental risk factors include ultraviolet (UV) exposure, tanning bed use, pregnancy, and chemical or carcinogen exposure [23,24,25,26]. Due to its strong genotoxic effects, melanoma development has been most commonly linked with UV radiation. Mutations caused by UV radiation account for >70% of the nucleotide mutations found in melanoma cases [27,28]. However, not all changes involved in melanoma incidence are UV-induced [29], and melanoma does not always occur in sun-exposed areas, a key difference from non-melanoma skin cancer [30,31]. Nearly one-third of melanoma cases are present in areas of the skin not usually exposed to UV light [31]. A small percentage of melanoma also occurs on mucosal surfaces, typically not exposed to the sun, and they tend to have a worse prognosis [32]. These findings suggest that UV exposure alone does not explain the sharply increased incidence of melanoma [7].

Pregnancy has been considered a trigger for melanoma since the 1950s [33,34]. A study of 1,309,501 maternities aged 15–44 years from 1994–2008 in New South Wales, Australia, found that the ratio of age-adjusted observed-to-expected rates for melanoma was 2.22 (95% CI = 2.05–2.41) [35,36]. While approximately one-third of melanoma cases in women are diagnosed during their childbearing age [37], these populations represent about 15% of melanoma cases. As melanoma incidence has increased similarly between males and females (1.90- and 1.81-fold increase from 2002 to 2022, respectively) [6,10], pregnancy alone does not explain the continued rise in melanoma cases. Other social-environmental risk factors such as older patients, organ transplant patients, and those with a history of immunosuppressive therapy are also conditions that have shown a significant correlation with aggressive melanoma, greater incidence of metastases, and lower survival rates [38]. These factors, however, are not modifiable.

Recent studies have shown links between melanoma incidence and other modifiable social-environmental factors such as obesity, tobacco use, and alcohol consumption. Obesity negatively impacts outcomes for surgically resected melanoma but leads to better outcomes when treated with immunotherapy [39,40]. However, the International Agency for Research on Cancer (IARC) found no evidence to correlate obesity and melanoma after reviewing more than 1000 epidemiological studies [41]. Linking tobacco use and increased risk of skin malignancies, especially melanoma, has been a topic of great investigation. However, the data remains unclear. While the IARC has declared smoking a cause of 18 cancers, cutaneous malignancies are not included in this data [42]. On the other hand, many studies have found a positive correlation between alcohol consumption and increased melanoma incidence, as discussed in Section 2.

This perspective review paper will summarize epidemiological data correlating melanoma incidence with alcohol consumption and discuss the potential roles of ethanol and ethanol metabolism in skin biology and melanoma biology. Understanding additional modifiable risk factors of melanoma would likely alter clinical management, patient education, and methods of public health outreach.

## 2. Alcohol and Melanoma

Accumulating evidence has suggested that alcohol consumption is positively linked to an increased risk of melanoma and that alcohol consumption is an independent risk factor for melanoma. This chapter will summarize the epidemiological data, including cohort and case-control studies and meta-analyses, and discuss the dose-dependent effects of alcohol and its associations with UV exposure.

### 2.1. Methods

**Figure 1:**
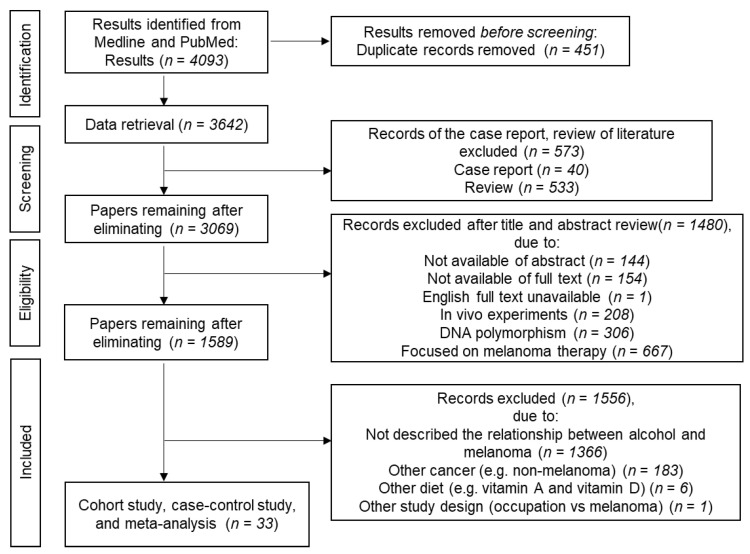
Flow diagram for collecting papers on alcohol drinking and melanoma risk.

We performed a scoping review on the association between alcohol and melanoma following the Preferred Reporting Items for Systematic Reviews and Meta-Analysis (PRISMA) extension for scoping reviews (PRISMA-ScR) guidelines (Supplemental Table S1) [43]. We excluded case reports, literature reviews, and others based on the criteria explained in the flow diagram (Figure 1).

#### 2.1.1. Literature Search

We conducted a literature search in Medline through Pubmed for epidemiological studies from 1977 to 29 July 2022. We designed the search strategy using the following keywords “melanoma” AND (“alcohol drinking” or “alcohol consumption” or “diet”) OR (“cohort study” or “case-control study” or “meta-analysis”) without restrictions on the geographic area, language, or publication status. The whole search is outlined in Supplemental Table S2.

#### 2.1.2. Study Selection and Data Extraction

Two authors (T.Y. and Z.Z.) independently screened the titles and abstracts of all retrieved records. Potentially relevant titles and abstracts were recorded, and the full-text articles were screened for final eligibility. Disagreements were resolved through discussion with a third author (M.F.).

### 2.2. Cohort and Case-Control Studies

The association between consumption and melanoma incidence was first suggested by Williams et al. in 1977 from the Third National Cancer Survey of 7518 patients with invasive cancer, including melanoma [44]. Subsequently, many cohort and case-control studies tried to examine the association [45,46,47,48,49,50,51,52,53,54,55,56,57,58,59,60,61,62,63,64,65,66,67,68,69,70,71,72,73]. Among 29 studies, 10 were cohort studies, and 19 were case-control studies. We listed each study’s findings on the alcohol and melanoma link in Table 1 and summarized these studies in Table 2. All studies assessed the effects of 1 or more types of alcohol (e.g., beer, wine, and spirits) on melanoma, and the intervention duration ranged from 2 to 60 years. Data from five studies investigated women only (Table 1, **#C6, C9, C21, C25, C27**). Alcohol consumption is positively correlated with melanoma incidence in 15 studies (**#C2, C4, C6, C8, C11, C13, C15, C18, C20, C23, C24, C25, C26, C28, C29**), negatively correlated in two studies (**#C9, C27**), and not correlated in 12 studies (**#C1, C3, C5, C7, C10, C12, C14, C16, C17, C19, C21, C22**).

Most studies assessed cutaneous but not non-cutaneous melanoma. Because non-cutaneous melanoma is not related to UV exposure, the potential risk factors of non-cutaneous melanoma may include other social-environmental risk factors such as alcohol consumption. However, no studies assessed the link between alcohol consumption and mucosal melanoma. Uveal melanoma was assessed only in #**C14**, where no association was found between alcohol consumption and uveal melanoma. More studies are needed to assess the relationship between non-cutaneous melanoma and alcohol consumption.

### 2.3. Meta-Analyses

While some cohort and case-control studies shown in Table 1 suggest an association between alcohol consumption and melanoma, the evidence regarding this link is inconsistent. Therefore, several research teams performed a meta-analysis of previously published data to assess the link quantitatively in larger numbers of melanoma cases and control groups. We identified four meta-analysis papers (Table 3).

The first report came from Rota et al. in 2014 (**#M1**), who showed that alcohol consumption was positively associated with melanoma risk (pooled relative risks (pRR) = 1.20; *p* = 0.006) by analyzing 14 case-control and two cohort studies of 6251 melanoma cases [74]. The following year, Miura et al. (**#M2**) reported a pooled analysis of eight melanoma case-control studies in women [75]. After analyzing 1,886 melanoma cases and 2113 controls, they found a positive association between melanoma and ever-consuming alcohol (adjusted pooled odds ratio (pOR) = 1.3; *p* < 0.05). Bagnardi et al. (**#M3**) investigated 572 studies on the effect of alcohol on 23 cancer types [76]. While cancer risks from alcohol were confirmed in the cancers of the oral cavity and pharynx, esophagus, colorectum, liver, larynx, and female breast, they found evidence of alcohol association in other cancers such as melanoma (RR = 1.11 for light drinkers and 1.20 for moderate drinkers). Interestingly, alcohol drinking was significantly associated with an increased risk of melanoma in studies conducted in North America (RR = 1.32 for light drinkers and 1.47 for moderate drinkers). Rivera et al. (**#M4**) used three large prospective cohort studies that followed 210,252 participants from the United States for 18.3 years [77]. They found that alcohol consumption was associated with an increased risk of aggressive melanoma (multivariate hazards ratio (HR) = 1.14 per drink per day; *P*_trend_ < 0.04). Interestingly, they also assessed 835 melanoma in situ cases and found a stronger association than invasive melanoma (multiariate HR = 1.46 per drink per day; *P*_trend_ < 0.0001). Recently, Mehta et al. reported a strong association between alcohol consumption and melanoma risk (pOR = 1.46; 95% CI = 1.32 –1.62; *p* < 0.00001) by analyzing five reports [78]. However, the study included one meta-analysis by Miura et al. (**#M2**), which weighted 52.7% of their study. Similarly, Gandini et al. analyzed 20 independent studies (10,555 melanoma cases and over 1.6 million non-cases/controls) and reported that alcohol intake was moderately associated with melanoma risk (summary relative risk (SRR) = 1.29 for the highest category vs. the lowest category of alcohol intake and SRR = 1.95 for cumulative intake) [79]. However, this study included a meta-analysis by Miura et al. (#**M2**), a meta-analysis by Rivera et al. (#**M4**), and a cohort study by Allen et al. (#**C21**) contributing to 80% of the controls; therefore, we excluded two meta-analyses studies by Metha et al. and Gandini et al. in Table 3.

Collectively, these four meta-analyses indicate a moderate detrimental effect of alcohol consumption on melanoma incidence.

### 2.4. Dose-Dependent Effects of Alcohol on Melanoma

Alcohol beverages can have both beneficial and detrimental impacts on human health, which could be influenced by ethanol amounts [80]. Therefore, the dose-specific effects of ethanol should be considered when discussing the detrimental effects of alcohol on melanoma incidence. Among 29 cohort and case-control studies, 20 studies included alcohol doses for their analysis (Table 1, **#C1, C2, C3, C4, C6, C10, C13, C14, C15, C16, C18, C19, C20, C21, C22, C23, C25, C26, C28, C29**). While seven studies showed no correlations (**#C1, C3, C10, C14, C19, C21, C22**), 13 studies clearly showed alcohol dose-dependent effects (**#C2, C4, C6, C13, C15, C16, C18, C20, C23, C25, C26, C28, C29**).

For example, Millen et al. (**#C15**) conducted a case-control study by recruiting 502 newly diagnosed patients with melanoma and 565 controls from outpatient clinics and computed ORs for melanoma and alcohol intake using logistic regression analysis [59]. Compared to crude OR (adjusted only for age, sex, and study site), adjusted OR (adjusted for not only age, sex, and study site, but also dysplastic nevi, education, and skin response to sun exposure such as tan vs. burn) became stronger (for example, 1.59 crude OR became 1.86 adjusted OR) and showed a dose-dependent effect trend for alcohol intake (% of kcal intake) (*P* for trend = 0.003). They also analyzed the relationship between melanoma and the number of alcoholic beverages and found that individuals who consumed ≥ 1.4 drinks per week had an increased risk for melanoma (OR = 1.55). While the data were not shown, they also mentioned that the magnitude of melanoma risk with increasing alcohol consumption was greater among women than men.

A prospective cohort study by Kubo et al. (**#C25**) followed 59,575 White postmenopausal women for over 10 years and examined the relationship between melanoma and alcohol consumption using Cox proportional hazards models [69]. Clinically important confounders were adjusted, such as age, education, smoking status and pack-year category, BMI category, physical activity, last medical visit, having insurance, having a care provider, history of non-melanoma skin cancer, history of melanoma, use of sunscreen, sun exposure, childhood and current summer sun exposure, and skin reaction to sun exposure. They found a significant relationship between melanoma and the amount of alcohol consumed, with those consuming seven or more drinks per week having the highest hazard of melanoma (HR = 1.64) compared to those consuming one to seven drinks per week (HR = 1.40) or less than one drink per week (HR = 1.10) (*P*_global_ = 0.0013). Lifetime alcohol consumption was also positively associated with melanoma risk (*p* = 0.0046).

A retrospective cohort study by Klatsky et al. (**#C26**) examined cancer incidence in 124,193 men and women from 1978 to 1985. They found that moderate (one to two drinks per day) or heavy (≥three drinks per day) alcohol drinking was associated with an increased risk of five cancers, including melanoma but not 12 other cancer types [70]. The HR of melanoma, adjusted for age, sex, race or ethnicity, body mass index, education, marital status, and smoking, was 1.9 (*p* < 0.001) or 2.2 (*p* < 0.001) for moderate or heavy drinkers, respectively, compared to lifelong alcohol abstainers. Interestingly, light drinking (<one drink per day) increased the risk of only two cancers: melanoma (HR = 1.6, 95% CI = 1.2–2.1, *p* < 0.01) and breast cancer (HR = 1.1, 95% CI = 1.0–1.2, *p* < 0.05).

Meta-analysis studies in Table 3 also addressed the association between alcohol doses and the risk of developing melanoma. Rota et al. (**#M1**) noted a dose-risk relationship between alcohol consumption and melanoma risk, with pRRs of melanoma of 1.10 for light drinking (≤1 drink/day = ≤12.5 g/day alcohol) and 1.18 for moderate-heavy alcohol drinking (>1 drink/day = >12.5 g/day alcohol) [74]. However, their meta-analysis could not shed light on the effect of high levels of alcohol intake, as little information was available on high alcohol doses and melanoma. Miura et al. (**#M2**) could not find a dose-response association from his pooled analysis [75]. The trend by Bagnardi et al. (**#M3**) is similar to that by Rota et al. While pRRs were 1.11 and 1.20 for light (≤12.5 g/day alcohol) and moderate (>12.5–≤50 g/day alcohol) drinkers, they could not evaluate the effect of heavy drinking (>50 g/day alcohol) on the risk of melanoma due to limited data [76].

On the other hand, Rivera et al. (**#M4**) conducted a combined dose-dependent analysis and showed an alcohol dose-dependent association with melanoma [77]. Compared with non-drinkers, the multivariate HRs of developing invasive melanoma were 1.14, 1.02, 1.21, and 1.24 for drinkers with increasing intake of alcohol (0.1–4.9, 5–9.9, 10–19.9, 20+ g/day, respectively). Interestingly, melanoma in situ showed a much stronger alcohol dose-dependent effect, with the multivariate HRs increasing with the increased intake of alcohol (1.13, 1.54, 1.75, and 1.57 for drinkers with alcohol intakes of 0.1–4.9, 5–9.9, 10–19.9, 20+ g/day, respectively). These results suggested that consuming one drink per day (12.8 g/day alcohol) increased the risk of invasive melanoma by 1.14-fold and melanoma in situ by 1.46-fold.

We also analyzed the World Health Organization (WHO) database globally and reported a strong positive correlation between alcohol consumption amount and melanoma incidence (*R* = 0.72; *p* < 0.001) by plotting each country’s data and calculating spearman’s rank correlation coefficient for melanoma incidence and mortality with alcohol consumption [81]. Interestingly, the data also revealed a positive correlation between alcohol dose and melanoma mortality (*R* = 0.59; *p* < 0.001).

Collectively, the reported studies suggest a positive relationship between alcohol dose and melanoma risk.

### 2.5. Effects of UV Exposure on the Association between Alcohol and Melanoma

Several studies provided the relationship between alcohol consumption and UV exposure, a significant risk factor for melanoma. Warthan et al. analyzed 56 sunburned beachgoers and reported that alcohol drinkers had a greater body surface area sunburned than non-drinkers (42% vs. 24%, *p* = 0.001), were more likely to develop blisters (31% vs. 5%, *p* = 0.02), and had a higher frequency of analgesic use after sunburn (69% vs. 26%, *p* = 0.007) [82]. Mukamal conducted a population-based telephone survey of 299,658 adults and reported that heavier alcohol use and binge drinking had more prevalence and number of sunburns than non-drinkers, with about 18% of sunburn cases associated with alcohol intake [83]. Therefore, to assess the detrimental effects of alcohol on melanoma incidence, we need to evaluate the impact of ethanol and UV exposure separately. Among 29 cohort and case-control studies, 12 studies took sun exposure into account (Table 1, **#C6, C8, C12, C13, C15, C16, C17, C18, C19, C20, C23, C25**). Among them, eight studies clearly showed UV-independent alcohol effects on melanoma (**#C6, C8, C13, C15, C18, C20, C23, C25**).

Meta-analysis studies in Table 3 also adjusted the association between alcohol and melanoma for UV exposure. Rota et al. (**#M1**) showed that the pRR of melanoma from alcohol drinking decreased from 1.25 to 1.15 after adjusting for UV exposure [74]. This data indicates a positive correlation between alcohol consumption and sun exposure, suggesting that heavy drinking may be associated with sun-seeking behaviors [59].

On the other hand, Miura et al. (**#M2**) demonstrated a positive correlation between alcohol intake and melanoma incidence after adjustment for sun exposure (pooled OR = 1.3; 95% CI = 1.1–1.5) [75]. They further assessed the influence of indoor tanning devices or sun exposure on alcohol-mediated melanoma risk and observed a statistically significant interaction between alcohol and never/ever-use of tanning devices (*P*_interaction_ = 0.043): Among those who never used tanning devices, alcohol consumption was positively associated with melanoma (OR = 1.38; 95% CI = 1.17–1.64, compared to ever users) whereas this association was not observed for those who ever used tanning devices (OR = 0.99; 95% CI = 0.72–1.33). They further assessed the influence of anatomic sites and found that alcohol drinkers have an increased risk for trunk melanoma (OR = 1.5; 95% CI = 1.1–1.9) but not other sites, such as head, neck, and extremities, which are more sun-exposed [75]. Rivera et al. (**#M4**) also showed a stronger association between alcohol consumption and melanoma in UV-protected skin (trunk) compared to UV-exposed skin (head, neck, and extremities) (1.73 vs. 1.03 HRs) [77].

In conclusion, many epidemiological studies demonstrated an association between alcohol consumption and melanoma. However, they do not necessarily indicate a causal relationship. The association seems to be alcohol dose-dependent and UV-independent, although alcohol and UV could work synergistically. Genetic predispositions and geographical influences may also add to the observed variance.

The following section will explore how alcohol consumption can contribute to melanoma development and progression.

## 3. Potential Roles of Ethanol on Melanoma Initiation and Progression

**Figure 2:**
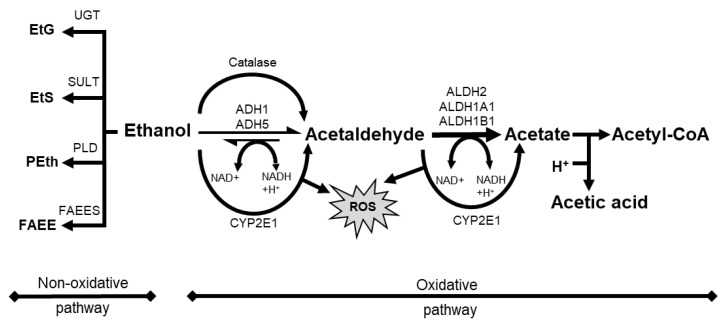
Schematic overview of alcohol metabolism with oxidative pathway and non-oxidative pathway. ADH, alcohol dehydrogenase; ALDH, aldehyde dehydrogenase; CYP2E1, cytochrome P 450 2E1; EtS, ethyl sulfate; FAEE, fatty acid ethyl ester; FAEES, fatty acid ethyl ester synthase; NAD, nicotinamide adenine dinucleotide; Peth, phosphatidyl ethanol; PLD, phospholipase D; ROS, reactive oxygen species; SULT, sulfotransferase; UGT, UDP-glucuronosyltransferase.

Absorption of orally administered alcohol beverages depends on ethanol concentration, blood flow, rate of ingestion and gastric emptying, beverage type, food intake, and the irritant properties of ethanol [84]. When ingested, ethanol is oxidized to toxic acetaldehyde (AcAH) by alcohol dehydrogenase (ADH) and then to acetic acid by mitochondrial aldehyde dehydrogenase 2 (ALDH2) (Figure 2, ethanol to a right direction) [84].

The putative benefits from alcohol intake come from various factors, including ethanol amount and ingredients [80]. For example, drinking small amounts of alcohol induces cardio-protective nitric oxide (NO) release in endothelial cells through ALDH2 activation [85]. Furthermore, non-ethanol active ingredients in alcoholic beverages (e.g., polyphenols) harbor anti-oxidant, anti-inflammatory, anti-carcinogenic, and other potential biological effects [86,87]. Rivera et al. [77] have found that white wine, but not red wine, was independently associated with an increased risk of cutaneous melanoma. A plausible explanation is that if the levels of AcAH in red and white wine are the same, the bioactive ingredients in red wine may offset its toxic effect.

However, the alcohol-mediated benefits disappear and are overridden by toxicity following heavy drinking and chronic consumption. In addition to the harmful intermediate metabolite AcAH, cytochrome p450 2E1 (CYP2E1) is induced and activated in response to high doses of ethanol, resulting in increased ROS (e.g., H_2_O_2_, hydroxide ion (OHˉ), and peroxide ion (O_2_ˉ)) [88]. IARC categorizes both ethanol and AcAH in alcoholic beverages as Group 1 carcinogens (carcinogenic to humans) [89]. Ethanol is associated with the tumor development of various cancers, such as liver and esophagus [76,90]. AcAH also induces GI tract tumors and lung cancer [91,92,93].

This chapter will review the roles of ethanol or AcAH in cellular biology and speculate on potential mechanisms connecting ethanol or AcAH to melanoma initiation and progression.

### 3.1. Roles of Ethanol or AcAH in Cellular Biology

Ethanol and/or AcAH induce oxidative stress, DNA damage, and lipid peroxidation, which activate protein kinases and signaling pathways implicated in glycolysis, fatty acid oxidation, inflammation, differentiation, angiogenesis, and metastasis, thereby creating a favorable microenvironment for tumor initiation and progression [94].

Both ethanol and AcAH promote oxidative stress. After ethanol uptake, ADH-catalyzed reactions in the cytosol and ALDH2-mediated reactions in the mitochondria reduce an NAD+/NADH redox ratio [84], regenerating NAD+ from NADH via the mitochondrial electron transfer system with concomitant ROS production [94].

AcAH is a highly reactive metabolite and a mutagen. AcAH-mediated DNA damage includes adduct formation, double-strand breaks, point mutations, DNA-DNA cross-links, sister chromatid exchanges, and chromosomal aberrations [95]. AcAH binds proteins involved in DNA repair and methylation, altering their structure and functions and promoting carcinogenesis [96,97,98]. AcAH also reacts with deoxyguanosine residues, leading to DNA modifications and lesions [99], impairing replication, transcription, and metabolism, and increasing mutation rates and cell death [99].

Another mutagenic effect of ethanol and/or AcAH is mediated by CYP2E1 induction, resulting in increased ROS generation. ROS-induced lipid peroxidation products such as malondialdehyde (MDA) and 4-hydroxynonenal (4-HNE) are genotoxic, thus generating mutagenic DNA adducts [100]. In addition, following ethanol intake, hybrid adducts can be generated in the affected tissues, such as the hybrid MDA and AcAH-protein adducts, increasing the tumorigenic potential of individual adducts by reducing NAD+ to NADH [100,101].

### 3.2. Roles of Ethanol or AcAH in Tumor Biology

**Figure 3:**
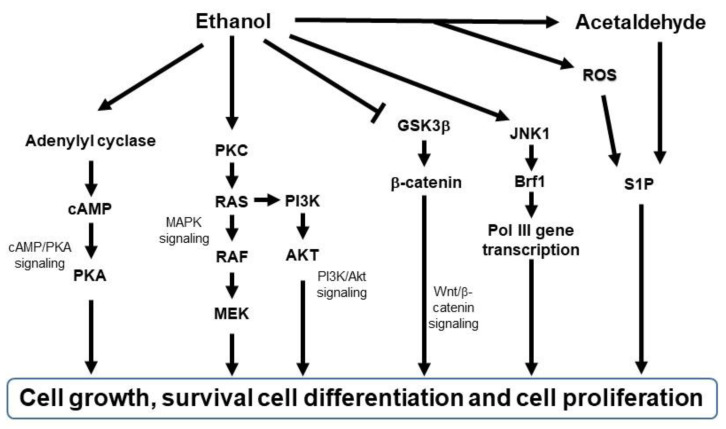
Schematic diagram of signaling pathways activated by ethanol. Brf1, TFIIB-related factor 1; AKT, protein kinase B; cAMP, cyclic adenosine monophosphate; GSK3β, glycogen synthase kinase 3 beta; JNK1, c-Jun N-terminal kinase; MAPK, mitogen-activated protein kinase; MEK, MAPK/Erk kinase; PI3K, phosphatidylinositol-3-kinase; PKA, protein kinase A; PKC, protein kinase C; RAF, rapidly accelerated fibrosarcoma; RAS, rat sarcoma virus; ROS, reactive oxygen species; S1P; sphingosine-1-phosphatase.

The abovementioned changes activate multiple signal transduction mechanisms, such as cAMP/PKA signaling [102,103,104], mitogen-activated protein kinase (MAPK) signaling [105], PI3K/Akt signaling [106,107], and Wnt/β-catenin signaling [108] (Figure 3). For example, ethanol stimulates cAMP-mediated PKA activation [104,109,110], and PKA activation has tumor-promoting or tumor-suppressive effects [111,112]. Ethanol also activates PKC [113]. Activated PKC induces RAS, RAF, or MEK 1/2 activation, leading to the activation of MAPK signaling to proliferate mammalian cells [114] and PI3K/Akt signaling to regulate cell survival and proliferation [115]. Furthermore, chronic alcohol consumption upregulates Wnt/β-catenin signaling, leading to tumor formation and progression in the liver cancer model [108] and tumor invasion in the colon cancer model [116]. Ethanol also induces JNK1-dependent upregulation of Brf1 expression and RNA Pol III gene transcription in breast cancer [117] and ethanol-induced liver cancer [118]. In addition, ethanol-induced genotoxic stress and oxidative stress can activate p53, which in turn activates sphingolipid-metabolizing enzymes, resulting in the accumulation of the ceramide metabolite sphingosine-1-phosphate (S1P), a promoter of the proliferation and inflammation in carcinogenesis [119]. These signaling pathways are key in initiating cellular responses implicated in tumorigenesis and progression, such as proliferation, differentiation, development, inflammation, survival, and cell death.

Lastly, ethanol and its metabolites damage various types of progenitor/stem cells, such as embryonic stem cells and tumor-initiating cells, impairing cell differentiation and genomic stability, leading to cellular aging and carcinogenesis [120,121].

### 3.3. Roles of Ethanol or AcAH in Skin Biology

Most research on alcohol consumption is centered around its effects on the liver and gastrointestinal tract but rarely on the skin [122,123]. Nonetheless, acute and chronic alcohol consumption induces various skin changes. Alcohol flush reaction is a typical acute response in which the rapid elevation of AcAH in the blood after drinking alcoholic beverages leads to erythema on the face, neck, and even the entire body [124], and this reaction occurs not only in ALDH2-deficient Asians but also in Caucasians and Native Americans [125,126]. Chronic alcohol consumption induces many skin changes such as jaundice, hyperpigmentation, and telangiectasis, which are often considered clinical manifestations of hepatic and vascular consequences [127,128].

However, it is crucial to consider ethanol’s direct effects on skin cells. For a long time, it has been known that ingested ethanol is secreted by the eccrine glands of human skin [129,130], with an almost equal concentration to blood concentration [131]. Ethanol can directly influence skin structure by disrupting skin cell membranes that form an effective barrier [132]. Furthermore, many skin microbiotas such as *Cutibacterium acnes*, *Staphylococcus aureus*, and *Staphylococcus epidermidis* possess ADH to convert ethanol to AcAH [133]. Therefore, it is likely that ethanol and AcAH exposure in the skin affects skin cell biology. The fact that chronic alcohol consumption causes esophageal melanosis in alcoholics [134,135,136] and skin hyperpigmentation in the epidermis of the paw and tail in mice [137,138,139] suggests that chronic alcohol ingestion promotes melanocyte changes.

In addition to oxidative metabolism, a smaller fraction of ethanol undergoes a non-oxidative route of metabolism (Figure 2, ethanol to a left direction). It results in the enzymatic conjugation of ethanol to endogenous metabolites, yielding ethyl glucuronide (EtG), ethyl sulfate (EtS), phosphatidylethanol (PEth), and fatty acid ethyl esters (FAEE) [140]. While only a minor fraction of total ethanol undergoes these metabolic pathways, the resulting metabolites such as EtG remain in the blood, urine, and hair for a long time (up to several months in hair). Therefore, these biometabolites are suitable biomarkers for recent alcohol use and abuse in clinical and forensic settings [141,142]. EtG and EtS are involved in toll-like receptor signaling, oxidative stress, and lower energy metabolism [143]. In contrast, PEth and FAEE interfere with cellular signaling pathways and disrupt organelle function [140]. Therefore, these biometabolites can also contribute to direct ethanol toxicity in organs with a limited oxidative capacity [140].

Furthermore, chronic alcohol consumption impairs skin immunology directly and/or indirectly by altering skin Langerhans cells [144,145], migrating dendritic cells [146], and multiple skin T cells [146].

Due to our use of alcohol-containing products and alcoholic beverages, these direct and indirect effects of ethanol and AcAH on our skin may not be easily eliminated. Together with the impact of skin microorganisms and potentially synergistic influences from sun exposure, these diverse effects on our skin may likely contribute to activating and transforming skin cells.

### 3.4. Does Ethanol or AcAH Affect Melanoma Initiation?

While mutagenic effects of ethanol and/or AcAH have been demonstrated in other cancers, their contribution to melanoma remains largely elusive.

Alcohol consumption lowers carotenoid levels in the plasma [147]. Carotenoids such as beta-carotene or lycopene can act as anti-oxidants to scavenge singlet molecular oxygen and peroxyl radicals generated during photo-oxidation and reduce solar light simulator-induced erythema [148]. AcAH is also a highly reactive chemical that serves as a photosensitizer [149]. Therefore, Darvin et al. hypothesized that alcohol consumption increased photosensitivity in human skin and recruited six male Caucasian volunteers [150]. They reported a decrease in the skin carotenoid concentration and minimal erythema dose (MED) after consuming 1 mL of ethanol/kg of body weight (corresponding to ~150 mL of vodka). However, these decreases were not observed after a combined intake of alcohol and ~1 liter of orange juice, rich in carotenoids. Low carotenoid levels increase erythema following UV exposure [148,151,152], and carotenoid consumption in the diet has been associated with decreased melanoma risk [59]. Therefore, these data suggest that alcohol consumption is associated with increased melanoma risk by lowering carotenoid levels and increasing UV sensitivity, indicating the synergistic effects of ethanol with UV exposure.

To elucidate the synergistic effects of ethanol and UV exposure on skin cells, Brand et al. used mouse models and human skin explants [153]. They demonstrated that combined ethanol consumption and UV exposure increased immune dysfunction and skin damage by decreasing DNA repair capacity and inhibiting protective mechanisms such as melanin production and anti-oxidants against UV exposure.

However, the mechanisms of melanoma development induced by UV light and ethanol are unclear. As mentioned in Section 3.1, excessive ethanol induces a complicated cellular response, from oxidative stress and persistent inflammation to mitochondrial DNA damage and signaling pathway activation [154,155], all implicated in tumor development. Among these pathways, MAPK signaling is one of the major pathways activated by mutations and is critical for melanoma initiation [156].

Strickland et al. reported that treating C3H/HeNCr mice with UV light and topical ethanol application (25% in water) thrice weekly for about 30 weeks induced primary cutaneous melanoma in 20 to 30% of the mice [157]. The frequency of melanoma induction was similar to that of squamous cell carcinoma. Topical ethanol application alone did not induce melanoma, and UV alone rarely induced melanoma. Interestingly, these melanoma tumors possessed *Nras* mutations at codons 13 and 19 in both tumors [158], which occurred at pyrimidine dimer sites, exhibiting a C to T transition on the non-transcribed strand at codon 13 and transcribed strand at codon 19, implicating UV-associated changes [159]. *BRAF* and *NRAS* are two major genes often mutated in human melanoma and are associated with melanoma initiation and progression [160]. Active *NRAS* mutations induce both MAPK and PI3K/Akt signaling [161]. Furthermore, these two mouse tumors and the cell lines had either a deletion in exon 2 of the *Ink4a*/*Arf* gene or an interstitial deletion of the long arm of chromosome 4 (where the *Ink4a*/*Arf* gene resides), similar to genetic changes of human melanoma for *CDKN2A*, encoding p16*^INK4a^* and p19*^ARF^* [162].

While UV is more frequently associated with the development of non-melanoma skin cancer than melanoma, it is unclear how the combination of ethanol and UV induced a relatively equal number of melanoma tumors compared to squamous cell carcinoma [157]. As ethanol stimulates cAMP-mediated PKA activation [104,109,110], this signaling may rewire β-catenin to activate the transcription of CREB target genes, including microphthalmia-associated transcriptional factor (MITF) [163], a master regulator of melanocyte biology [164,165]. Alterations in the MITF gene and pathway are associated with a higher risk of melanoma initiation [166,167]. MITF is regulated by several other transcription factors, including SOX10, CREB, Pax3, Tyro3, and TCF/LEF. The activation of the BRAF V600E/ERK pathway can also enhance the expression of MITF by directly phosphorylating MITF at Ser73 or activating CREB through MSK [164,168,169,170]. The PI3K/Akt signaling pathway regulates MITF through inactivating GSK-3β or cooperating with RAS/RAF/MEK/ERK signaling. In addition, MITF is a target of the p38/MAPK signaling but can be inhibited by the JNK/MAPK pathway, suggesting that the regulation of MITF is accomplished by various MAPK signaling pathways [171,172].

Considering these data collectively, it is likely that such signaling pathways are critical components of ethanol-induced carcinogenesis in some cancers, including melanoma.

### 3.5. Does Ethanol or AcAH Affect Melanoma Progression?

Several studies have reported the biological effects of chronic alcohol consumption on melanoma progression and metastasis. Tan et al. found that ethanol-treated B16F10 melanoma tumors exhibited enhanced angiogenesis through increased vascular endothelial growth factor (VEGF) expression, contributing to tumor progression [173]. On the other hand, Meadow’s research team from Washington State University studied alcohol’s impact on tumor metastasis in C57BL/6 mice using B16BL6 melanoma, a derivative of B16F10 with a more invasive and metastatic phenotype [174,175,176,177]. Melanoma cells were injected subcutaneously to assess spontaneous metastasis and intravenously to assess experimental metastasis. While pretreating tumor cells in vitro or in vivo with ethanol enhanced experimental metastasis [174], pretreating mice with ethanol (10–20% (*w/v*) ethanol for >4 weeks) inhibited spontaneous and experimental metastases [174,177]. These data suggest that ethanol directly potentiates the metastatic capacity of melanoma cells. They also indicate that the host environment at the tumor injection determines the ethanol’s effect on tumor metastasis. Interestingly, survival times were significantly shorter in mice pretreated with ethanol, despite having fewer metastases [174], implicating the detrimental effects of ethanol in tumor-bearing mice.

Several mechanisms could be involved in alcohol-mediated melanoma progression and metastasis. For example, ethanol disturbs mitochondrial dynamics [178] by increasing mitochondrial fission and reducing their fusion [179], and altered mitochondrial dynamics can promote tumor migration and progression in human melanoma [180]. ROS from mitochondria upregulates hypoxia-inducible factor-1α, inducing matrix metalloproteinases and VEGF [181], important for tumor invasion. Furthermore, DNA and ATP leaked from damaged mitochondria activate inflammasomes [181,182], reshaping the tumor microenvironment and immune infiltration to support tumor progression and drug resistance, as shown in our previous reports [183,184,185].

Another consequence of chronic and acute alcohol consumption is interference with immune cell numbers and function, which may facilitate melanoma progression and metastasis [163,186]. B16BL6 melanoma-bearing mice exposed to chronic ethanol showed fewer mature B cells, CD8+ T cells, and NK cells in circulation due to ethanol-induced downregulation of S1P/S1P receptor 1 signaling resulting in decreased egress of lymphocytes from the spleen [187]. Chronic ethanol administration also impairs the trafficking of NK cells to lymph nodes, resulting in a decreased number and percentage of NK cells in the draining nodes [188]. CD8+ T cells and NK cells are important in inhibiting tumor progression. Moreover, chronic ethanol exposure in mice impairs antigen-specific response to melanoma cells by inhibiting the proliferation of memory T cells, reducing IFN-γ producing CD8+ T cells, and increasing myeloid-derived suppressor cells [189].

Ethanol can also upregulate the expression of nerve growth factor receptor (NGFR/CD271) through NF-κB signaling in human melanoma cells [190]. NGFR expression in melanoma cells is associated with increased metastasis and long-term growth [191,192]. NGFR signaling also plays a critical role in acquired melanoma resistance to BRAF/MEK inhibitors [193,194,195,196]. VEGF expression, which contributed to angiogenesis in the melanoma mice model, is also reported to induce immune resistance by affecting myeloid-derived suppressor cells, dendritic cells, T regulatory cells, and cytotoxic T cells [173,197]. In addition, NGFR expression induced by ethanol is linked to immunosuppressive functions and anti-PD-1 immunotherapy resistance in melanoma [198,199,200].

Together, the accumulated data suggest a link between chronic alcohol consumption and melanoma progression. Further studies are required to ascertain these findings, especially in human melanoma patients, and elucidate the underlying mechanisms and biology. In addition to the factors mentioned above, the role of other potential factors such as genetic instability, metabolic rewiring, and skin microbiota need to be determined for a comprehensive understanding of alcohol-associated melanoma progression.

## 4. Conclusions

Despite greater public health and UV protective efforts, melanoma incidence has continued to increase, warranting further exploration into alternative, modifiable social-environmental risk factors that may be implicated in melanoma pathogenesis. In addition to UV exposure, ethanol and AcAH have each been found to cause genetic changes associated with carcinogenesis. Specifically, ethanol and AcAH’s carcinogenic activity is tied to their ability to induce various forms of DNA damage and signaling activation. However, the particular role of these adducts and pathways in melanoma initiation remains unclear. Recent epidemiology studies revealed a positive correlation between alcohol and melanoma incidence. It seems clear that chronic alcohol drinking produces persistent and complex changes in skin cell components, metabolic activities, and signaling networks that lead to a progressive pathological transition in physiological responses. Equally important, chronic alcohol consumption leads to disturbances in ethanol metabolism, an inflammatory milieu, and an immunosuppressive state that contributes to melanoma initiation, progression, and metastasis. These effects of alcohol could be synergistic with or independent of UV exposure. Understanding the mechanism underlying the causal relationship between alcohol consumption and melanoma initiation is still in its infancy. Further research is needed to determine the roles of ethanol and AcAH in melanoma development.

## Figures and Tables

**Table 1:** Literature review of cohort and case-control studies on alcohol drinking and melanoma.

Study #	Authors (Year)	Study Design	No. (MM)	No. (Control)	Alcohol Consumption	Risk Ratio	95% CI	*p* (Single or Trend *)	Correlation to Alcohol	Ref
**C1**	Green et al. (1986)	case-control	91	89	Any	RR = 0.74	0.39–1.40		null	[45]
					≤1 drink/day	RR = 0.70	0.24–2.04			
					>1 drink/day	RR = 0.76	0.34–1.70			
**C2**	Holman et al. (1986)	case-control	511	511	Any	RR = 1.37	1.09–1.71		positive	[46]
					≤1 drink/day	RR = 1.16	0.79–1.71			
					>1 drink/day	RR = 1.48	1.13–1.95			
**C3**	Osterlind et al. (1988)	case-control	474	924	Any	RR = 0.75	0.57–0.99		null	[47]
					≤1 drink/day	RR = 0.80	0.59–1.08			
					>1 drink/day	RR = 0.69	0.50–0.96			
**C4**	Stryker et al. (1990)	case-control	204	248	10 g/day	OR = 1.8	1.0–3.3	0.03	positive	[48]
**C5**	Adami et al. (1992)	cohort	10,350 (individuals)		Any	SIR = 0.9	0.3–1.9	N/A	null	[49]
**C6**	Bain et al. (1993)	case-control	41 (women)	297 (women)	0.1–9.9 g/day	OR = 0.78	0.32–1.94	0.12 (trend)	positive	[50]
					10.0–19.9 g/day	OR = 1.40	0.46–4.3			
					≥20 g/day	OR = 2.5	0.87–7.4			
**C7**	Kirkpatrick et al. (1994)	case-control	234	248	Any	RR = 1.23	0.72–2.12		null	[51]
**C8**	Bataille et al. (1996)	case-control	255	253	Any	pOR = 2.5	1.7–3.7	N/A	positive	[52]
**C9**	Sigvardsson et al. (1996)	cohort	15,508 (women)		Registered alcoholics	RR = 0.5	0.3–1.0	N/A	negative	[53]
**C10**	Wesrerdahl et al. (1996)	case-control	400	640	1–9 g/day	OR = 0.8	0.6–1.1	>0.05 (trend)	null	[54]
					10–19 g/day	OR = 0.9	0.5–1.5			
					≥20 g/day	OR = 0.9	0.5–1.8			
**C11**	Rolon et al. (1997)	case-control (male)	41 (plantar MM)	168 (hospital control)	Current or ex-drinkers	OR = 2.5	1.3–5.1	N/A	positive	[55]
**C12**	Veierod et al. (1997)	cohort	25,708 (men) 25,049 (women)		Beer	IRR = 0.7	0.3–1.4	N/A	null	[56]
					Wine/liquor	IRR = 0.6	0.3–1.2			
					Beer	IRR = 1.4	0.6–3.4			
					Wine/liquor	IRR = 1.7	0.9–3.2			
**C13**	Freedman et al. (2003)	cohort	68,588 (white)		<1–6 drinks/week	RR = 1.2	0.8–1.8	0.08 (trend)	positive	[57]
					7–14 drinks/week	RR = 1.4	0.8–2.5			
					>14 drinks/week	RR = 2.1	0.9–4.8			
**C14**	Stang et al. (2003)	case-control	118	475	1–15 g/day	OR = 1.0	0.5–1.8		null	[58]
					16–27 g/day	OR = 0.7	0.3–1.4			
					>28 g/day	OR = 1.0	0.5–2.1			
**C15**	Millen et al. (2004)	case-control	502	565	0.2–1 % kcal	OR = 0.97	0.62–1.50	0.003 (trend)	positive	[59]
					1–4 % kcal	OR = 1.16	0.76–1.77			
					4–10 % kcal	OR = 1.86 ^§^	1.24–2.78			
					≥10 % kcal	OR = 1.65 ^§^	1.09–2.49			
					0.7 drinks/week	OR = 1.04	0.69–1.57	0.04 (trend)	positive	
					1.4–7.0 drinks/week	OR = 1.55 ^§^	1.09–2.20			
					7.7–59 drinks/week	OR = 1.53 ^§^	1.03–2.29			
**C16**	Naldi et al. (2004)	case-control	542	538	<1 drinks/week	OR = 0.81	0.53–1.22	N/A	null	[60]
					1–13 drinks/week	OR = 0.91	0.62–1.33			
					14–27 drinks/week	OR = 1.26	0.83–1.91			
					≥28 drinks/week	OR = 0.83	0.49–1.40			
**C17**	Vinceti et al. (2004)	case-control	59	59	Energy-adjusted tertiles	RR = 1.86	0.64–5.42	0.978	null	[61]
**C18**	Le Marchand et al. (2006)	case-control	177 (males)	177 (males)	45,421–265,001 g/lifetime ≥265,002 g/lifetime	OR = 1.2	0.6–2.2	0.01 (trend)	positive (male) null (female)	[62]
						OR = 2.3	1.2–4.4			
			111 (females)	111 (females)	45,421–265,001 g/lifetime	OR = 1.1	0.5–2.4	0.19 (trend)		
					≥265,002 g/lifetime	OR = 1.7	0.7–3.8			
**C19**	Fortes et al. (2008)	case-control	304	305	Wine (weekly)	OR = 1.28	0.80–2.04	0.73 (trend)	null	[63]
					(daily and more)	OR = 0.83	0.49–1.42			
					Exclusive wine (weekly)	OR = 0.79	0.34–1.84	0.36 (trend)	null	
					(daily and more)	OR = 0.64	0.22–1.88			
					Beer (less than weekly)	OR = 1.05	0.67–1.64	0.99 (trend)	null	
					(more than weekly)	OR = 0.98	0.53–1.79			
					Spirits	OR = 1.15	0.72–1.83		null	
**C20**	Gogas et al. (2008)	case-control	55	165	>1 drink/day	OR = 2.45	1.00–6.13	0.05	positive	[64]
**C21**	Allen et al. (2009)	cohort	1,280,296 (middle-aged women)		<2 drinks/week	RR = 1.0	0.93–1.07	0.3 (trend)	null	[65]
					3–6 drinks/week	RR = 1.0	0.92–1.08			
					7–14 drinks/week	RR = 0.96	0.88–1.05			
					>15 drinks/week	RR = 1.17	1.00–1.37			
**C22**	Benedetti et al. (2009)	case-control	107	507	1–6/week	OR = 0.93	0.50–1.72	N/A	null	[66]
					7+/week	OR = 1.21	0.68–2.18			
					7+/wk (0–71 drinks/yr)	OR = 1.32	0.69–2.52	0.586 (trend)	null	
					7+/wk (72–179 drinks/yr)	OR = 0.71	0.31–1.63			
					7+/wk (180+ drinks/yr)	OR = 1.65	0.71–3.83			
**C23**	Asgari et al. (2012)	cohort	69,635 (individuals)		<1 drink/day	HR = 1.19	0.96–1.48	0.05 (trend)	positive	[67]
					1–1.9 drinks/day	HR = 1.33	1.01–1.76			
					≥2 drinks/day	HR = 1.28	0.97–1.70			
**C24**	de Vries et al. (2012)	case-control	360	1550	Regular alcohol consumption	OR = 1.32	1.01–1.74	0.04	positive	[68]
**C25**	Kubo et al. (2014)	cohort	59,575 (white women)		Past drinker	HR = 0.99	0.64–1.52	0.0013 (trend)	positive	[69]
					<1 drink/month	HR = 0.88	0.55–1.40			
					<1 drink/week	HR = 1.10	0.74–1.66			
					1–7 drinks/week	HR = 1.40	0.95–2.06			
					≥7 drinks/ week	HR = 1.64	1.09–2.49			
					>0–5 drink-yr/lifetime	HR = 1.35	0.99–1.83	0.0046 (trend)	positive	
					>5–10 drink-yr/lifetime	HR = 1.66	1.19–2.33			
					>10–20 drink-yr/lifetime	HR = 1.55	1.09–2.21			
					>20–50 drink-yr/lifetime	HR = 1.79	1.29–2.50			
					>50–200 drink-yr/lifetime	HR = 1.98	1.32–2.95			
**C26**	Klatsky et al. (2015)	cohort	124,193 (individuals)		Ex-drinker	HR = 1.4	0.9–2.2			[70]
					<1 drink/day	HR = 1.6 ^§^	1.2–2.1	< 0.01	positive	
					1–2 drinks/day	HR = 1.9 ^§^	1.4–2.6	< 0.001	positive	
					≥3 drinks/day	HR = 2.2 ^§^	1.6–3.1	< 0.001	positive	
**C27**	Mahamat-Saleh et al. (2019)	cohort	404	67,332	Any (median 7.9 g/day, mostly wine))	HR = 0.89	0.73–1.09		negative	[71]
			(Women)							
**C28**	Malagoli et al. (2019)	case-control	380	719	Red wine (2nd tertile)	OR = 0.94 ^†^	0.64–1.36		null	[72]
					Red wine (3rd tertile)	OR = 0.83 ^†^	0.58–1.19		null	
					White wine (2nd tertile)	OR = 1.44 ^†^	1.01–2.06		positive	
					White wine (3rd tertile)	OR = 1.03 ^†^	0.73–1.45		null	
					Aperitif wines and beer (2nd tertile)	OR = 0.94 ^†^	0.66–1.36		null	
					Aperitif wines and beer (3rd tertile)	OR = 0.83 ^†^	0.58–1.19		null	
					Spirits and liqueurs (2nd tertile)	OR = 0.93 ^†^	0.64–1.36		null	
					Spirits and liqueurs (3rd tertile)	OR = 0.92 ^†^	0.63–1.35		null	
**C29**	Sanford et al. (2020)	cohort	3100 (MM patients)		Current drinking	OR = 1.59	1.45–1.75		positive	[73]
					Exceeding moderate drinking	OR = 0.95	0.84–1.08			
					Binge drinking	OR = 1.20	1.05–1.38		positive	

* *p* for trend: across quintile medians in the adjusted model. ^§^ *p* < 0.05. † vs. 1st tertile. Abbreviation. CI, confidence interval; HR, hazard ratio; IRR, incidence rate ratios; MM, malignant melanoma; N/A, not applicable; OR, odds ratio; pOR, pooled odds ratio; RR, relative risks; SIR, Standardized incidence rate.

**Table 2:** Summary of cohort and case-control studies.

	Cohort Study (*n* = 10)	Case-Control Study (*n* = 19)
**Melanoma correlation**	10/10 papers	19/19 papers
** Positive**	4 (40%)	10 (52.6%)
** Negative**	2 (20%)	0 (0%)
** Null**	3 (30%)	9 (47.4%)
**Dose-dependent effects**	6/10 papers	14/19 papers
** Positive**	5 (83.3%)	8 (57.1%)
** Negative**	0 (0%)	0 (0%)
** Null**	1 (12.7%)	6 (42.9%)
**Link to UV/Sun exposure**	4/10 papers	8/19 papers
** Positive**	3 (75.0%)	5 (62.5%)
** Negative**	0 (0%)	0 (0%)
** Null**	1 (25.0%)	3 (37.5%)

**Table 3:** Literature review of meta-analysis studies on alcohol drinking and melanoma.

Study #	Authors (Year)	Study Cases and Types	No. (MM)	No. (Control)	Alcohol Consumption	Risk Ratio	95% CI	*p* (Single or Trend *)	Ref
**M1**	Rota et al. (2014)	16 studies (2 cohort and 14 case-control studies)	6251	N/A	Any (case-control, 14)	pRR = 1.2	1.01–1.44	0.003	[74]
					Any (cohort, 2)	pRR = 1.26	1.19–1.35	0.657	
					Any (overall, 16)	pRR = 1.2	1.06–1.37	0.006	
					≤1 drink (≤12.5 g) /day	pRR = 1.10	0.96–1.26	0.045	
					>1 drink (≥12.5 g) /day	pRR = 1.18	1.01–1.40	0.021	
**M2**	Miura et al. (2015)	8 case-control studies	1886	2113	Any	pOR = 1.30	1.1–1.5	<0.05	[75]
					0.5–<3.5 g/day	pOR = 1.30	1.0–1.7		
					3.5–<6.8 g/day	pOR = 1.30	1.0–1.7		
					6.8–<14.4 g/day	pOR = 1.30	0.9–1.7		
					14.4–127.3 g/day	pOR = 1.0	0.7–1.3		
**M3**	Bagnardi et al. (2015)	14 studies (2 cohort and 12 case-control studies)	4631	1465	≤12.5 g/day	pRR = 1.11	0.97–1.27	0.156	[76]
					>12.5–≤50 g/day	pRR = 1.20	1.03–1.41		
					>50 g/day	n.e.			
**M4**	Rivera et al. (2016)	3 prospective cohort studies	1374 Invasive MM	N/A	0.1–4.9 g/day	mHR ^a^ = 1.13	0.91–1.41	0.04 (trend)	[77]
					5–9.9 g/day	mHR ^a^ = 1.02	0.81–1.28		
					10–19.9 g/day	mHR ^a^ = 1.21	0.97–1.49		
					20+ g/day	mHR ^a^ = 1.23	0.96–1.59		
					Per drink (12.8 g)/day	mHR ^a^ = 1.14	1.00–1.29		
			835 MIS		0.1–4.9 g/day	mRR ^a^ = 1.13	1.19–2.00	<0.0001 (trend)	
					5–9.9 g/day	mRR ^a^ = 1.54	0.81–1.28		
					10–19.9 g/day	mRR ^a^ = 1.75	1.14–2.70		
					20+ g/day	mRR ^a^ = 1.57	1.12–2.22		
					Per drink (12.8 g)/day	mRR ^a^ = 1.46	1.24–1.72		

^a^ mHR and mRR: adjusted for age, BMI, smoking status, physical activity, caffeine intake, family history of melanoma, tanning ability, lifetime number of severe sunburns, number of moles on forearms, hair color at age 18, and average annual UV-B flux at place of residence. Abbreviation. CI, confidence interval; mHR, multivariate hazard ratio; MM, malignant melanoma; MIS, melanoma in situ; N/A, not applicable; n.e., not evaluable; OR, odds ratio; pOR, pooled odds ratio; mRR, multivariate relative risk; pRR, pooled relative risk; SOR, summary odds ratio. * *p* for trend: across quintile medians in the adjusted model.

## Supplementary Materials

The following supporting information can be downloaded at: https://www.mdpi.com/article/10.3390/cancers14205010/s1. Table S1: Preferred Reporting Items for Systematic reviews and Meta-Analyses extension for Scoping Reviews (PRISMA-ScR) Checklist. Table S2: Search strategy.
