# Supplementary material for: Alcohol as a Non-UV Social-Environmental Risk Factor for Melanoma
Source: Cancers (Basel). 2022 Oct 13;14(20):5010. doi: 10.3390/cancers14205010 (PMC9599745; doi:10.3390/cancers14205010)
Supplement: Supplementary file 1 [file cancers-14-05010-s001.zip › Supplemental table S2 Search strategy 081722.pdf]

Search strategy

We will conduct a literature search in Medline through Pubmed for epidemiological studies and will use the following search string: [“melanoma” AND (alcohol drinking OR alcohol consumption OR diet) OR (cohort study OR case-control study OR meta-analysis)], which comprises the following medical subject heading terms: ‘alcohol’, ‘diet’ and ‘melanoma’.

1. melanoma AND alcohol drinking
2. melanoma AND alcohol consumption
3. melanoma AND diet
4. melanoma AND cohort study
5. melanoma AND case-control study
6. melanoma AND meta-analysis
7. melanoma AND alcohol drinking AND cohort study
8. melanoma AND alcohol drinking AND case-control study
9. melanoma AND alcohol drinking AND meta-analysis
10. melanoma AND alcohol consumption AND cohort study
11. melanoma AND alcohol consumption AND case-control study
12. melanoma AND alcohol consumption AND meta-analysis
13. melanoma AND meta-analysis AND cohort study
14. melanoma AND meta-analysis AND case-control study
15. melanoma AND meta-analysis AND meta-analysis study
